# Natural history and clinical burden of moderate aortic stenosis: a systematic review and explorative meta-analysis

**DOI:** 10.2459/JCM.0000000000001490

**Published:** 2023-06-26

**Authors:** Martina Morelli, Michele Galasso, Giuseppe Esposito, Francesco Stefano Soriano, Stefano Nava, Caterina Da Pozzo, Irene Bossi, Emanuela Piccaluga, Giuseppe Bruschi, Alessandro Maloberti, Fabrizio Oliva, Jacopo Andrea Oreglia, Cristina Giannattasio, Claudio Montalto

**Affiliations:** a School of Medicine and Surgery, University of Milano-Bicocca; b Interventional Cardiology, 1^st^ Division of Cardiology, De Gasperis Cardio Center, Niguarda Hospital; c School of Medicine and Surgery, University of Milan; d Department of Cardiac Surgery; e 4^th^ Division of Cardiology, De Gasperis Cardio Center, Niguarda Hospital, Milan, Italy

**Keywords:** meta-analysis, moderate aortic stenosis, prognosis, systematic review, valvulopathy

## Abstract

**Aims:**

The mortality risk of patients with moderate aortic stenosis is not well known, but recent studies suggested that it might negatively affect prognosis. We aimed to assess the natural history and clinical burden of moderate aortic stenosis and to investigate the interaction of patients’ baseline characteristics with prognosis.

**Methods:**

Systematic research was conducted on PubMed. The inclusion criteria were inclusion of patients with moderate aortic stenosis; and report of the survival at 1-year follow-up (minimum). Incidence ratios related to all-cause mortality in patients and controls of each study were estimated and then pooled using a fixed effects model. All patients with mild aortic stenosis or without aortic stenosis were considered controls. Meta-regression analysis was performed to assess the impact of left ventricular ejection fraction and age on the prognosis of patients with moderate aortic stenosis.

**Results:**

Fifteen studies and 11 596 patients with moderate aortic stenosis were included. All-cause mortality was significantly higher among patients with moderate aortic stenosis than in controls in all timeframes analysed (all *P* < 0.0001). Left ventricular ejection fraction and sex did not significantly impact on the prognosis of patients with moderate aortic stenosis (*P* = 0.4584 and *P* = 0.5792), while increasing age showed a significant interaction with mortality (estimate = 0.0067; 95% confidence interval: 0.0007–0.0127; *P* = 0.0323).

**Conclusion:**

Moderate aortic stenosis is associated with reduced survival. Further studies are necessary to confirm the prognostic impact of this valvulopathy and the possible benefit of aortic valve replacement.

## Introduction

Aortic stenosis is the most common valvulopathy in Europe and North America, and its prevalence is increasing with ageing.^1,2^ In fact, nowadays, calcific aortic valve disease (also known as degenerative or senile) is the most common cause of aortic stenosis in high-income countries.^3,4^ According to current guidelines, moderate aortic stenosis (mAS) is defined as an aortic valve area between 1 and 1.5 cm^2^, a mean pressure gradient between 20 and 40 mmHg or a peak aortic jet velocity between 3 and 4 m/s.^5^ Several pivotal trials revealed that severe aortic stenosis negatively affects prognosis,^6,7^ and current guidelines recommend early treatment with either transcatheter aortic valve implantation (TAVI) or surgical aortic valve replacement (SAVR).^1,5^ On the contrary, the impact of mAS has not been extensively studied, and ongoing trials are aiming at assessing if its early treatment is associated with improved outcomes.^8^ The aim of the present systematic research and meta-analysis is to explore the natural history and clinical burden of mAS and to investigate the interaction of baseline characteristics with the prognosis of patients with this valvular heart disease.

## Materials and methods

### Search strategy

A systematic literature search of PubMed was conducted in February 2022, using the following search algorithms: (“Aortic Valve Stenosis”[Mesh]) AND (moderate[Title/Abstract]) AND ((“Prognosis”[Mesh]) OR (“Mortality”[Mesh]) OR (“Survival”[Mesh]) OR (“Disease Progression”[Mesh])) and (moderate aortic stenosis[Title/Abstract]) AND ((outcome[Title/Abstract]) OR (mortality[Title/Abstract]) OR (survival[Title/Abstract]) OR (prognosis[Title/Abstract]) OR (natural history[Title/Abstract]) OR (disease progression[Title/Abstract])). The following filters were applied: human species and Italian, English, French and German language. Articles were screened by title and abstract content. In addition, the reference lists of all eligible studies were screened.

Articles were considered eligible if they fulfilled the following criteria: inclusion of patients with mAS; and report of the survival at 1-year follow-up (minimum).

For each study, the following information was extracted: publication data (first author, journal, publication year), study design (number of patients and controls included, subpopulations considered), population characteristics (including age, sex, BMI, comorbidities, cardiovascular risk factors, ongoing therapies, NYHA class), echocardiographic characteristics [including left ventricular ejection fraction (LVEF), mean pressure gradient, peak aortic jet velocity, aortic valve area] and outcome data [including mortality, survival, aortic stenosis progression, aortic valve replacement (AVR)]. Data extraction was performed by two authors (M.M., M.G.) in parallel; all controversies were examined with a senior author (C.M.).

### Statistical analysis

Continuous variables were expressed as mean ± standard deviation and categorical variables as count (%). When data were available only as median and interquartile range, mean and standard deviation were calculated according to Wan *et al.*^9^ Baseline characteristics from each study were pooled and compared between patients with mAS and controls, obtaining pooled weighted means with 95% confidence intervals (CIs). Incidence ratios related to all-cause mortality in patients and controls of each study with 95% CIs were estimated from raw mortality data and then pooled using the fixed effects Mantel–Haenszel model.^10^ Meta-regression analysis was performed to assess the impact of LVEF, female sex and age on the prognosis of patients with mAS. Statistical heterogeneity was estimated by calculating the *I*^2^ index. Heterogeneity was considered to be low for *I*^2^ less than 25%, moderate for *I*^2^ 25–75% and high for *I*^2^ more than 75%.^11^ Individuals without aortic stenosis or with mild aortic stenosis were defined as controls. Statistical significance was set at a two-sided *P*-value less than 0.05. Data analysis was performed in the R environment.

## Results

**Fig. 1:**
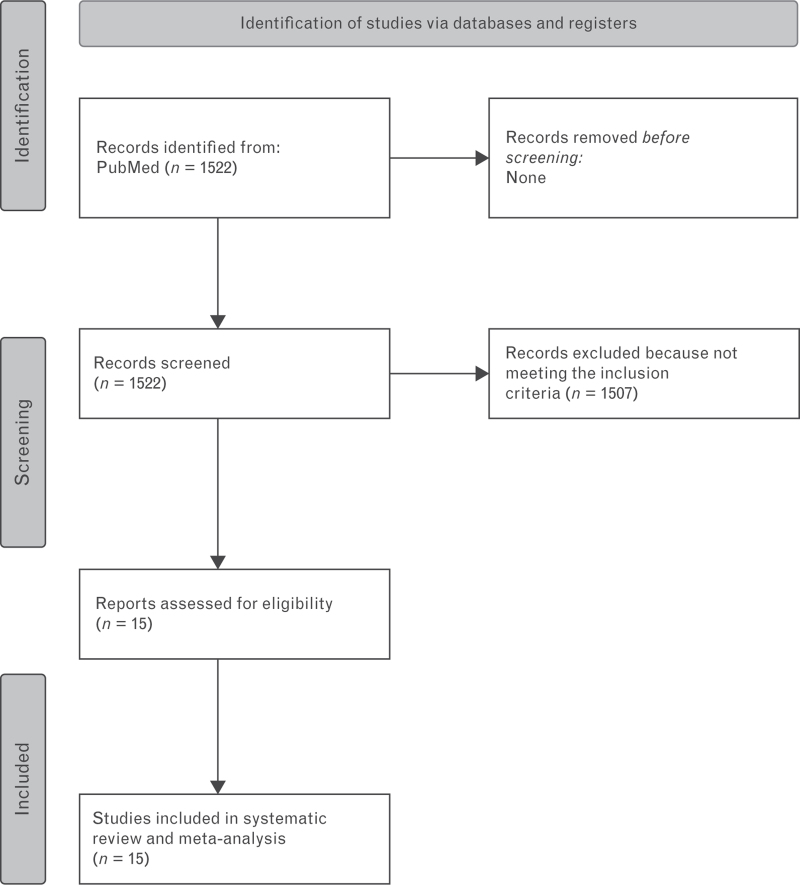
PRISMA flow diagram.

The systematic literature search identified 1522 studies. Fifteen articles were included in this systematic review with a total of 11 596 patients with mAS (Fig. 1 and Table 1). These studies were observational with a mean follow-up ranging from 1.8 to 9.5 years. They studied the prognosis, assessing all-cause mortality, cardiovascular mortality, AVR, aortic stenosis progression, complications, hospitalization for heart failure, progression of symptoms.

**Table 1:** Studies included

Ref.	Journal	Year of publication	*n* Patients	Controls	Mean follow-up (years)
Coisne *et al.*^12^	JAMA Cardiology	2021	1122	Mild AS	2.1
Mann *et al.*^13^	Valvular Heart Disease	2021	952	Non-moderate	3.5
Du *et al.*^14^	BMC Cardiovascular Disorders	2021	729	/	5.0
Chew *et al.*^15^	American Journal of Cardiology	2021	738	/	9.5
Jean *et al.*^16^	Journal of the American College of Cardiology	2021	262	No AS	2.9
Lee at al.^17^	Journal of Korean Medical Science	2021	787	General population	7.7
Bae *et al.*^18^	Heart Surgery Forum	2020	148	/	5.6
Delesalle *et al.*^19^	American Heart Association	2019	508	/	3.9
Strange *et al.*^20^	Journal of the American College of Cardiology	2019	3315	No AS and mild AS	3.3
Van Gils *et al.*^21^	Journal of the American College of Cardiology	2017	305	/	4.0
Romero *et al.*^22^	American Journal of Cardiology	2014	2358	Mild AS	2.3
Yechoor *et al.*^23^	Journal of Thoracic and Cardiovascular Surgery	2013	104	/	1.8
Rosenhek *et al.*^24^	European Heart Journal	2004	176	/	4.0
Iivanainen *et al.*^25^	American Journal of Cardiology	1996	26	No AS and mild AS	4.0
Kennedy *et al.*^26^	Journal of the American College of Cardiology	1991	66	/	2.9

Patients with mAS had a mean age of 75.7 years (95% CI: 75.53–75.85) and were predominantly male (Table 2). The prevalence of cardiovascular risk factors was high with 31.2% (95% CI: 0.30–0.32) of patients affected by diabetes, 66.2% (95% CI: 0.57–0.76) by hypertension and 38.0% (95% CI: 0.36–0.40) by dyslipidaemia. These patients had also important comorbidities: 17.9% (95% CI: 0.17–0.19) of patients presented chronic kidney disease, 25.7% (95% CI: 0.25–0.27) coronary artery disease, 19.4% (95% CI: 0.18–0.21) atrial fibrillation, 7.7% (95% CI: 0.07–0.08) previous stroke and 5.9% (95% CI: 0.05–0.07) peripheral artery disease. Patients with mAS were also frequently on cardiovascular therapy, including beta blockers [49.8% (95% CI: 0.48–0.52)], renin-angiotensin-aldosterone system inhibitors [47.0% (95% CI: 0.45–0.49)], statins [56.5% (95% CI: 0.54–0.59)], antiplatelets [50.4% (95% CI: 0.48–0.53)] and oral anti-coagulants [26.1% (95% CI: 0.24–0.28)]. Mean aortic valve area of patients with mAS was 1.3 cm^2^ (95% CI: 1.25–1.26), mean peak aortic jet velocity was 3.2 m/s (95% CI: 3.17–3.19) and mean LVEF was 61.5% (95% CI: 61.36–61.70) (Table 2).

**Table 2:** Pooled characteristics of patients with moderate aortic stenosis and controls

Baseline characteristics	Moderate AS	Mild or no AS	*P*
Female sex (%)	30.42 (0.29–0.31)	48.55 (0.48–0.49)	<0.0001
Diabetes (%)	31.18 (0.30–0.32)	17.56 (0.17–0.18)	<0.0001
Hypertension (%)	66.22 (0.57–0.76)	60.79 (0.34–0.87)	<0.0001
Dyslipidaemia (%)	38.04 (0.36–0.40)	25.68 (0.25–0.26)	<0.0001
Smoke (%)	5.29 (0.05–0.06)	8.16 (0.08–0.08)	<0.0001
Chronic kidney disease (%)	17.93 (0.17–0.19)	4.73 (0.05–0.05)	<0.0001
Coronary artery disease (%)	25.67 (0.25–0.27)	16.09 (0.14–0.18)	<0.0001
Atrial fibrillation (%)	19.44 (0.18–0.21)	14.95 (0.13–0.17)	<0.0001
Previous stroke (%)	7.67 (0.07–0.08)	1.95 (0.02–0.02)	<0.0001
Peripheral artery disease (%)	5.89 (0.05–0.07)	6.64 (0.05–0.08)	0.1139
NYHA 3–4 (%)	0.29 (0.00–0.00)	10.49 (0.09–0.12)	0.0002
Beta blocker (%)	49.80 (0.48–0.52)	52.31 (0.49–0.56)	<0.0001
RAS inhibitor (%)	46.98 (0.45–0.49)	70.18 (0.66–0.75)	<0.0001
Statin (%)	56.46 (0.54–0.59)	61.21 (0.57–0.65)	0.0158
Antiplatelet (%)	50.39 (0.48–0.53)	45.06 (0.41–0.49)	0.0187
Oral anticoagulant (%)	26.08 (0.24–0.28)	25.41 (0.23–0.28)	0.0255
Age (years)	75.69 (75.53–75.85)	61.74 (61.67–61.80)	<0.0001
BMI (kg/m^2^)	26.84 (26.71–26.98)	26.87 (26.84–26.90)	0.7338
Valve area (cm^2^)	1.26 (1.25–1.26)	1.90 (1.90–1.91)	<0.0001
Ejection fraction (%)	61.53 (61.36–61.70)	60.33 (60.29–60.37)	<0.0001
Peak jet velocity (m/s)	3.18 (3.17–3.19)	1.49 (1.48–1.49)	<0.0001

### Outcomes

**Fig. 2:**
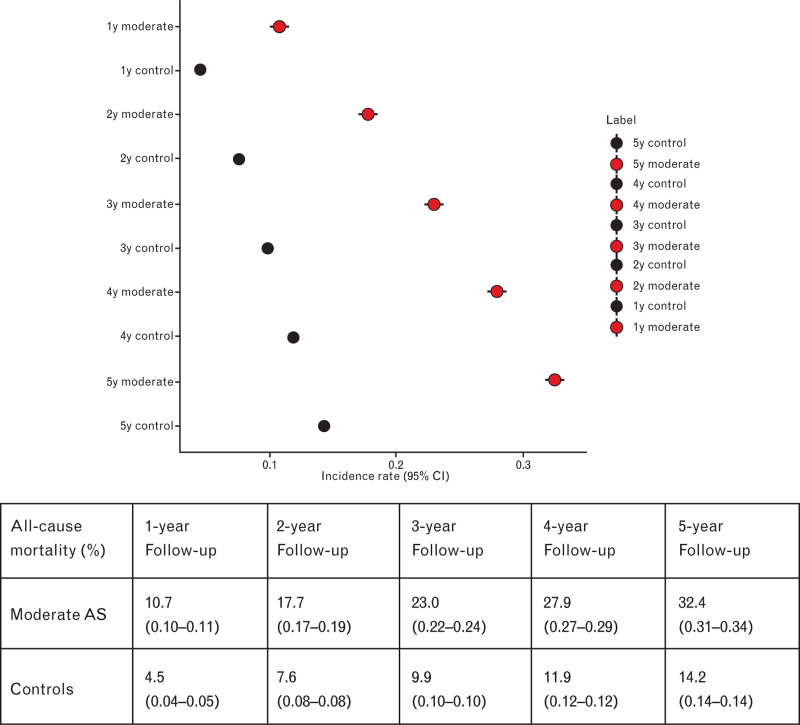
All-cause mortality of patients with moderate aortic stenosis (red) versus controls (black) at 1-, 2-, 3-, 4- and 5-year follow-up.

All-cause mortality was assessed among patients with mAS and among controls (which included mild and no aortic stenosis). All-cause mortality was significantly higher in those with mAS than among controls at each timepoint analysed (Fig. 2) and it ranged from 10.7% (95% CI: 0.10–0.11) versus 4.5% (95% CI: 0.04–0.05) at 1-year follow-up (*P* < 0.0001) to 32.4% (95% CI: 0.31–0.34) versus 14.2% (95% CI: 0.14–0.14) at 5-year follow-up (*P* < 0.0001) (Supplementary Figures 1–5). Heterogeneity was high for all timepoints analysed.

**Fig. 3:**
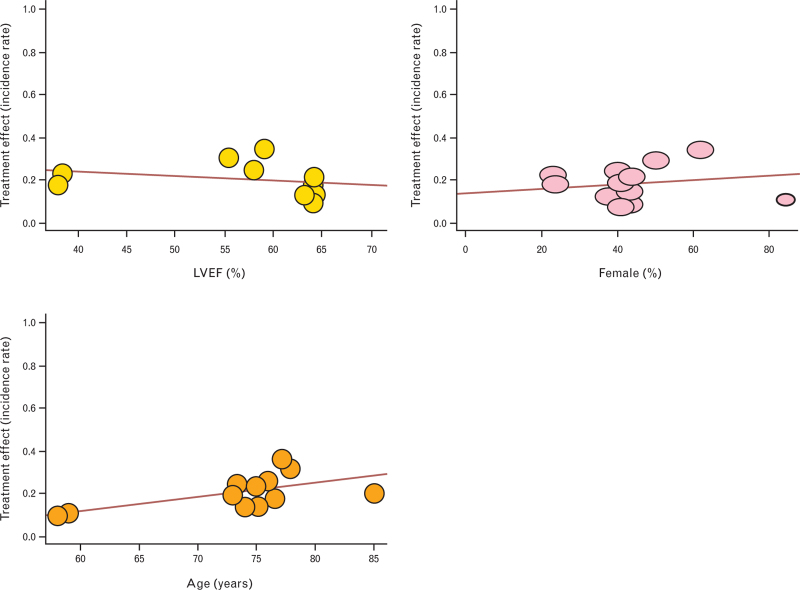
Impact of left ventricular ejection fraction (yellow) and age (orange) on prognosis of patients with moderate aortic stenosis.

A meta-regression analysis was performed and suggested that neither LVEF nor sex significantly impacted on the prognosis of patients with mAS (estimate = −0.0020; 95% CI: −0.0078 to 0.0038; *P* = 0.4584 for LVEF; estimate = −0.0010; 95% CI: −0.0029 to 0.0049; *P* = 0.5792 for female sex); on the contrary, age was associated with a significantly worse prognosis of these patients (estimate = 0.0067; 95% CI: 0.0007–0.0127; *P* = 0.0323) (Fig. 3).

## Discussion

Aortic stenosis is a common disease; it is progressive nature and potentially fatal when it becomes severe. Therefore, it is recommended that physicians should screen patients and treat them early when symptoms develop, in order to avoid excess mortality. Both European and American guidelines for the management of valvular heart diseases mostly focus on severe aortic stenosis with indications for TAVI or SAVR mainly driven by age and comorbidities.^1,5^ Nonetheless, several small recent studies individually showed that mAS (even if asymptomatic) might negatively affect prognosis,^12–26^ although a precise estimate of the mortality risk associated with mAS is unknown. Some older studies revealed that mAS is relatively benign, but the definition of the disease and the population affected have changed over time.^27,28^

Our systematic review shows that mAS is not a benign disease, as these patients experience a worse prognosis compared with those with mild aortic stenosis or no aortic stenosis. In particular, we also offer a tentative estimate of the excess mortality risk, with a relative risk for death ranging from 1.43 at 1-year to 2.28 at 5-year follow-up. In fact, despite including a large number of patients in our analysis, we acknowledge that the observational nature of the studies included generates a large heterogeneity that limits the external validity of our meta-analysis, the results of which should then be interpreted as explorative. Nonetheless, our results are in line with those of another recent meta-analysis, which showed that mAS appears to be associated with an excess mortality risk, which is expectedly lower than severe aortic stenosis.^29^ In addition to this, we explored possible interactions with other important characteristics and showed that neither LVEF nor sex significantly impacts on mortality at mid-term. This is important, as both have been associated with worse prognosis in severe aortic stenosis^30,31^ but, in the context of mAS, close follow-up and early treatment, regardless of baseline characteristics, appear to be of prominent importance. Only age was associated with a significantly worse prognosis, which is biologically expected and reinforces the internal validity of our analysis.

In summary, our data highlight some important concepts about mAS. Firstly, mAS is not a benign disease and it is intrinsically associated with a higher hazard of death. In particular, the relative increase of death at 1 year suggests that mAS might have an intrinsic potential risk including rapid progression to severe aortic stenosis with early fatal outcomes. We also postulate that in some cases, especially those with low-flow low-gradient aortic stenosis due to impaired LVEF, a truly severe aortic stenosis might be erroneously downgraded to mAS and treated conservatively, despite a clear indication for intervention. The idea of aortic stenosis as a spectrum of hazard that ranges from mAS to asymptomatic severe aortic stenosis to symptomatic severe aortic stenosis is being tested also in ongoing randomized and controlled clinical trials such as the EARLY-TAVR trial, which is evaluating the TAVI on patients with asymptomatic severe aortic stenosis,^32^ and the TAVR UNLOAD trial, which is testing TAVI on patients with mAS and heart failure with reduced LVEF.^8^ Our results corroborate this hypothesis and highlight that we should not neglect mAS and at least screen it for severity and symptoms.

Secondly, one could argue that, if the procedural risk is relatively low as with TAVI, a lower threshold for intervention could be applied in selected cases. This is corroborated by some retrospective studies, which showed that the AVR significantly improved the survival of patients with mAS, especially in those with impaired LVEF.^16,19,22,33^ Nonetheless, it should be highlighted that, if considered TAVI candidates, lifetime management considerations will be of prominent importance, as these individuals are younger (mean age: 75.7 years) and not free from coronary artery disease (prevalence: 25.7%). Moreover, careful preprocedural screening is anticipated, as complications such as paravalvular leaks and conduction disturbances leading to permanent pacemaker implantations could not be tolerated in younger and more active patients with mAS (or asymptomatic severe aortic stenosis). Furthermore, also, valve deterioration and consequent need for a second (and even third) procedure during the individual's lifespan should be taken into consideration, especially in younger patients, who could outlive their transcatheter heart valve, as their life expectancy could exceed the durability of the valve bioprosthesis.^34^

### Study limitations

Some limitations should be taken into consideration. First, the systematic literature search was conducted on PubMed only. Second, this work included only observational studies, due to the lack of clinical trials on this topic, which limits the generalizability of our findings. This is also testified by the high heterogeneity of our meta-analysis results, which should therefore be regarded as explorative and hypothesis-generating only. Third, some mortality rates were not explained in the text of the articles, so these data were extrapolated from Kaplan--Meier plots. Fourth, Supplementary Figures 1–5 show that pooled all-cause mortality of patients with severe aortic stenosis is lower than that of patients with mAS. This result is unexpected and not biologically plausible and it deserves interpretation: firstly, the literature search was focused on mAS so it should not be interpreted as complete for severe aortic stenosis; secondly, due to the current management of this valvulopathy, patients with untreated mAS were compared with patients with treated severe aortic stenosis, which might account for a better prognosis in the latter group, and even reinforce our previous considerations. Finally, patients with mAS and controls differed from each other significantly: it is possible that there was a confounder associated with this valvulopathy.

## Conclusion

Moderate aortic stenosis is associated with a higher all-cause mortality risk; therefore, it might deserve close follow-up and further studies are necessary to confirm its prognostic importance and possible benefits of early intervention.

### Conflicts of interest

There are no conflicts of interest.

## Supplementary Material

Supplemental Digital Content
