# Supplementary material for: Natural history and clinical burden of moderate aortic stenosis: a systematic review and explorative meta-analysis
Source: J Cardiovasc Med (Hagerstown). 2023 Jun 26;24(9):659–65. doi: 10.2459/JCM.0000000000001490 (PMC10754483; doi:10.2459/JCM.0000000000001490)
Supplement: Supplemental Digital Content [file jcarm-24-659-s001.docx]

**Supplementary figure 1.** *All-cause mortality at 1-year follow-up.*

**Supplementary figure 2.** *All-cause mortality at 2-year follow-up.*

**Supplementary figure 3.** *All-cause mortality at 3-year follow-up.*

**Supplementary figure 4.** *All-cause mortality at 4-year follow-up.*

**Supplementary figure 5.** *All-cause mortality at 5-year follow-up.*
